# Exome Sequencing Identifies Susceptibility Loci for Sarcoidosis Prognosis

**DOI:** 10.3389/fimmu.2019.02964

**Published:** 2019-12-24

**Authors:** Elisa Lahtela, Matti Kankainen, Juha Sinisalo, Olof Selroos, Marja-Liisa Lokki

**Affiliations:** ^1^Transplantation Laboratory, Department of Pathology, University of Helsinki, Helsinki, Finland; ^2^Institute for Molecular Medicine Finland, University of Helsinki, Helsinki, Finland; ^3^Medical and Clinical Genetics, Helsinki University Hospital, University of Hesinki, Helsinki, Finland; ^4^Heart and Lung Center, University of Helsinki and Helsinki University Hospital, Helsinki, Finland; ^5^University of Helsinki, Helsinki, Finland

**Keywords:** sarcoidosis, prognosis, MHC, whole exome sequencing, 1p36.21, leucocyte receptor complex

## Abstract

Many sarcoidosis-associating immunological genes have been shown to be shared between other immune-mediated diseases. In Finnish sarcoidosis patients, good prognosis subjects more commonly have HLA-DRB1*03:01 and/or HLA-DRB1*04:01-DPB1*04:01 haplotype, but no marker for persistent disease have been found. The objective was to further pinpoint genetic differences between prognosis subgroups in relation to the HLA markers. Whole-exome sequencing was conducted for 72 patients selected based on disease activity (resolved disease, *n* = 36; persistent disease, *n* = 36). Both groups were further divided by the HLA markers (one/both markers, *n* = 18; neither of the markers, *n* = 18). The Finnish exome data from the Genome Aggregation Database was used as a control population in the WES sample. Statistical analyses included single-variant analysis for common variants and gene level analysis for rare variants. We attempted to replicate associated variants in 181 Finnish sarcoidosis patients and 150 controls. An association was found in chromosome 1p36.21 (AADACL3 and C1orf158), which has recently been associated with sarcoidosis in another WES study. In our study, variations in these genes were associated with resolved disease (AADACL3, *p* = 0.0001 and *p* = 0.0003; C1orf158, *p* = 7.03E-05). Another interesting chromosomal region also peaked, Leucocyte Receptor Complex in 19q13.42, but the association diminished in the replication sample. In conclusion, this WES study supports the previously found association in the region 1p36.21. Furthermore, a novel to sarcoidosis region was found, but additional studies are warranted to verify this association.

## Introduction

Sarcoidosis is granulomatous disease characterized by the presence of non-caseating granulomas in affected organs. Sarcoidosis can affect any organ in the body and the disease course can be self-limited or chronic (1). These different outcomes have led to the classification of sarcoidosis based on prognosis: resolved disease, i.e., duration of disease <2 years and persistent disease with a longer duration (≥2 years) (2).

Exact etiology of sarcoidosis is still unclear. The heterogeneity of sarcoidosis in clinical course and organ involvement has led to the hypothesis that sarcoidosis might not be one disease, but consists of several disease entities, each with distinct genetic associations (3). This may explain why no single sarcoidosis-associated variant has been found, but a wide range of genes with relatively small effects. With a current knowledge it seems, that the genetic susceptibility to sarcoidosis is mainly affected by the genetic variation in the genes and pathways related to granuloma formation and immune response. The MHC region in chromosome 6p21.3 contains multiple genes essential for the immune system. The association for sarcoidosis in the MHC have been found in all classes through I to III (4, 5). In our previous studies in Finnish patients, an association between class II HLA-DRB1*03:01 and the haplotype consisting of HLA-DRB1*04:01-DPB1*04:01 and good prognosis compared to poor prognosis was found (44.9 vs. 22.7%; *p* = 0.001; OR = 2.78; 95% CI = 1.45–5.24) (6, 7).

Besides the MHC, other susceptible sarcoidosis risk/protective chromosome regions and genes have been found throughout the genome. Beside classical candidate-gene approaches, genome-wide association analyses (GWAS) have become method of choice nowadays. However, as sarcoidosis is a rare disease, its prevalence in Finland being 28 per 100,000 (8), collections of large case-control materials for GWAS are demanding. A possible new method for finding causality in genetics behind sarcoidosis is whole-exome sequencing (WES). The exome sequences encompass only about 2% of the human genome but harbors about 85% of all described disease-causing variants (9), making smaller sample sizes sufficient for identification of novel genes.

Aim of this study was to further characterize genetic differences between Finnish sarcoidosis prognosis utilizing whole-exome sequencing method in the subset of 72 patients and to replicate the findings in a bigger data set of 188 Finnish patients. In Finnish patients, subjects with good prognosis are more likely to have above mentioned class II HLA markers, but no genetic markers have been found for persistent disease. The objective was to further pinpoint genetic variety of sarcoidosis prognosis in relation to the HLA markers.

## Materials and Methods

### Study Subjects and Selection Criteria

Study subjects and their characteristics have been previously described (7). In summary, a total of 188 Finnish patients with verified pulmonary sarcoidosis had been followed-up for 5–15 years (Supplementary Table 1). After follow-up the patients were clinically divided into those with disease resolved within 2 years (*n* = 90) and to those with persisting activity after 2 years (*n* = 98). Disease activity was evaluated using the generally accepted WASOG (World Association of Sarcoidosis and Other Granulomatous diseases) criteria (10). The clinical examinations included a chest radiograph, a lung function test (spirometry, diffusion capacity), electrocardiography (ECG), liver enzymes, serum calcium, creatinine, serum lysozyme, and serum ACE.

**Figure 1:**
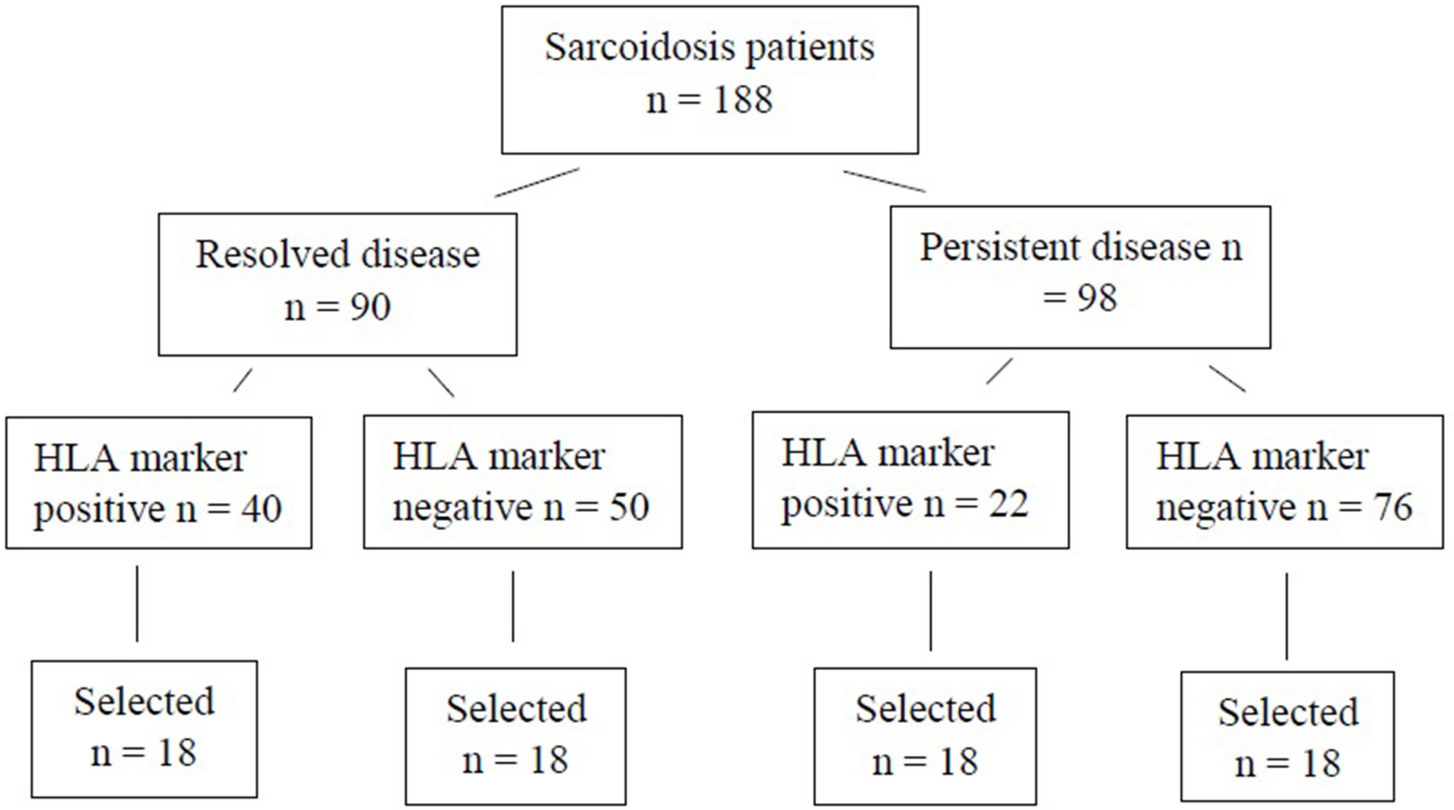
Finnish sarcoidosis patients. Selection criteria based on sarcoidosis prognosis and the presence or absence of the HLA markers (HLA-DRB1*03:01 and/or HLA-DRB1*04:01-DPB1*04:01 haplotype). From each of the HLA positive/negative groups, 18 samples were randomly selected for this study.

For the WES study, a subset of 72 patients were chosen (Figure 1). Patients were selected based on disease activity (resolved disease, *n* = 36; persistent disease, *n* = 36). These subsets were further divided by the HLA markers previously known to influence disease prognosis in Finnish patients (HLA-DRB1*03:01 and HLA-DRB1*04:01-DPB1*04:01) yielding in 18 patients with one or both markers (HLA+) and 18 patients with neither of the markers (HLA–). Same selection was used in both resolved and persistent patient groups. The Genome Aggregation Database (gnomAD) (11) was used as control population in the WES study. GnomAD database consists of exome data from 11 150 Finnish subjects, with no known diseases.

For the replication of the SNPs found in the WES study, the rest of the original above mentioned 188 Finnish sarcoidosis patients were genotyped. A control population of 150 healthy subjects representing the Finnish population (12) was included for the replication phase.

The study protocol was approved by the Ethics Committee of the Department of Internal Medicine, Hospital District of Helsinki and Uusimaa, Helsinki, Finland. All subjects provided written informed consent for their participation in genetic association studies.

### Whole Exome Sequencing

The DNA extraction have been previously explained (7). The WES was performed in Institute for Molecular Medicine Finland (FIMM). 150 ng of gDNA was fragmented with Covaris E220 evolution instrument (Covaris, Woburn, MA, USA). Sample libraries and MedExome Enrichment were processed according to SeqCapEZ HyperCap Workflow User's Guide (Roche Nimblegen, Madison, WI, USA). Enrichment was performed in 4 samples Multiplexed DNA Sample Library Pools using 1 μg of each library. The amplified library was purified with 1.8x Agencourt AMPure XP beads and eluted to 200 μl EB-buffer. Library was quantified for sequencing using 2100 Bioanalyzer High sensitivity kit. Sequencing was performed with Illumina HiSeq2500 system in HiSeq High Output mode using v4 kits (Illumina, San Diego, CA, USA). Read length for the paired-end run was 2 × 101 bp. 91% of all samples had the target coverage of 20x.

### Variant Calling

Sequencing data were pre-processed using Trimmomatic (leading:3, trailing:3, sliding window:4:15, illuminaclip:2:30:10, minlen:36). Paired-end reads passing the filtering were mapped to the reference genome (GRCh38) using the BWA-MEM algorithm. Reads were sorted by coordinate and duplicates were marked with the Picard tools (http://broadinstitute.github.io/picard/). Base qualities were recalibrated and local indel realignment performed around indels at the BAM-level using Genome Analysis Toolkit (GATK) followed by merging of data by sample and remarking of duplicates with the Picard tools. Joint genotyping was performed on the whole cohort of 72 samples using GATK HaplotypeCaller and variants were recalibrated using GATK VariantRecalibrator. GATK tools were applied as recommended by GATK guidelines and using GATK resource files that had been converted from GRCh37 to GRCh38 using CrossMap and chain files from EnsEMBL. Quality control analysis of sequencing data was performed using the FastQC (http://www.bioinformatics.babraham.ac.uk/projects/fastqc) with default settings. Additionally, frameshift indels were excluded due to recognized difficulties calling from WES data (13).

Annotation and filtering of variants was performed using the Annovar tool against the RefGene database. Filtering quality control was as follows: coverage >10, variant quality value >40, frequency <1%, and assumed to impair protein function. The mean coverage of exomic regions for each variant was 43X with individual variant reads varying between 17 and 153X. Reads were manually checked using Integrative Genomics Viewer (IGV). Variants with unknown frequency in Finnish population in the gnomAD database were excluded. Version information and references of tools used in variant calling are given in Supplementary Table 2.

### Testing for Genetic Association

We analyzed associations for single variants and variant groups within genes. The associations were evaluated between all the dichotomous groups (persistent vs. resolved disease, HLA+ persistent vs. resolved disease, and HLA- persistent vs. resolved disease). For single-variant analysis, the common variants [minor allele frequency (MAF) > 0.01] were included. The hypergeometric distribution method in EPACTS software pipeline (https://genome.sph.umich.edu/wiki/EPACTS) was used. The gene-based tests were assessed to find whether variants in each gene are jointly associated with the phenotype. Gene-based association improves the power to detect rare variants (MAF < 0.05) in limited sample size. Two methods were used to test the associations: hypergeometric distribution and SKAT-O (14) methods implemented in the EPACTS software. SKAT-O test was used, because it assumes that each variant can have either positive or negative effect to the phenotype. The *p* were corrected for multiple comparisons (number of studied variants) by using False Discovery Rate (FDR) method and Bonferroni correction. A value of *p* < 0.05 was considered statistically significant. Age, gender, and extrapulmonary manifestations were used as covariates in all statistical tests. To assess the functionality of the found variations in protein level, we used Sift (15) and PolyPhen (16) databases, which both predict possible impact of an amino acid substitution on the structure and function of a human protein using straightforward physical and comparative considerations. To further investigate the possibly functional effects of the significant SNPs, we used the Genotype-Tissue Expression (GTEx) Portal (17) to study the expression quantitative trait loci (eQTL).

For the replication, the SNPs that reached the significant association level in single-variant and gene-based analysis were included (Supplementary Table 3). The SNP genotyping was performed with the Agena Bioscience (Sequenom) MassARRAY System (Agena Biosciences, San Diego, California) at the Institute for Molecular Medicine Finland (FIMM), Helsinki, Finland, with standard protocols. Genotypes were called using Sequenom's MassARRAY Typer software. The allele frequencies between different groups were compared by a case-control association analysis (Chi-square χ^2^ test, PLINK software) (18). We applied the following quality control filters: minimum call rate per sample of 90%, SNP minor allele frequency (MAF) > 0.01 and Hardy Weinberg equilibrium (HWE) > 0.001. Total success rate for accepted SNP arrays was 95% in the replication samples.

## Results

### Single-Variant Analysis

**Figure 2:**
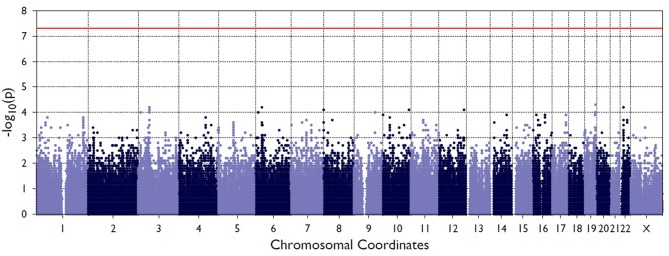
Manhattan plot from the single-variant association tests between persistent and resolved patients. The X-axis shows chromosome position the Y-axis shows the negative log of *p*-values so that higher values represent stronger significance levels. The horizontal red line shows the threshold value for significance.

Figure 2 shows the Manhattan plot from the single-variant association tests between resolved and persistent patients showing the highest associating peak in the chromosome 19, although not reaching the exome-significance level. Supplementary Figure 1 shows the quantile-quantile (Q-Q) plot for the single-variant tests, which demonstrates the similarity between observed and expected significance values. Table 1 shows the single variants that yielded the strongest associations in analyses between resolved and persistent, and between HLA+ and HLA– prognosis groups. In analysis between resolved and persistent patients altogether seven variants associated with the prognosis. Interestingly, five of these variants were located in two chromosomal regions: three in chromosome 1 (1p36.21) and two in chromosome 19 (19q13.42). In HLA+ group, the associations in these two chromosomal locations remained. In HLA- patients the chromosomal locations 1p36.21 and 19q13.42 did not associate with the prognosis. None of these associations remained significant after correcting for multiple testing with FDR or Bonferroni correction.

**Table 1:** Associated variations in single-variant analysis in different Finnish sarcoidosis subgroups based on disease prognosis (persistent, resolved) and HLA markers (HLA+ = HLA–DRB1^*^03:01 and/or HLA-DRB1^*^04:01-DPB1^*^04:01; HLA– = without either of the markers).

**Chromosome**	**Position**	**Chromosomal location**	**MAF Persistent**	**MAF Resolved**	**MAF gnomAD Finnish**	**SNP**	**AA Change**	**Predicted Function**	***P***	**rsID**	**Gene**
**PERSISTENT VS. RESOLVED**											
**1**	**12719616**	**1p36.21**	**0.125**	**0.375**	**0.2196**	**C/T**	**Ser47Pro**	**B/T**	**0.000915**	**rs3010877**	***AADACL3***
**1**	**12725527**	**1p36.21**	**0.181**	**0.389**	**0.3072**	**T/G**	**Cys125Phe**	**P/T**	**0.016591**	**rs7513079**	***AADACL3***
**1**	**12760937**	**1p36.21**	**0.139**	**0.361**	**0.2346**	**C/T**	**Phe153Leu**	**B/T**	**0.00833**	**rs1132185**	***C1orf158***
1	156267524	1q22	0.000	0.069	0.01	T/C	Ile355Val	B/T	0.0269443	rs145100575	*SMG5*
**19**	**54664811**	**19q13.42**	**0.208**	**0.375**	**0.3021**	**G/A**	**Asp223Gly**	**B/D**	**0.01645**	**rs731170**	***LILRB4***
**19**	**54818581**	**19q13.42**	**0.264**	**0.153**	**0**	**G/A**	**Met113Val**	**B/D**	**0.046766**	**rs643861**	***KIR3DL1, KIR3DS1***
20	35434589	20q11.22	0.444	0.347	0.3917	C/A	Glu144Ala	B/D	0.0397467	rs224331	*GDF5*
**HLA+**											
**1**	**12719616**	**1p36.21**	**0.083**	**0.361**	**0.2196**	**C/T**	**Ser47Pro**	**B/T**	**0.001023**	**rs3010877**	***AADACL3***
**1**	**12725527**	**1p36.21**	**0.111**	**0.333**	**0.3072**	**T/G**	**Cys125Phe**	**P/T**	**0.008796**	**rs7513079**	***AADACL3***
**1**	**12760937**	**1p36.21**	**0.111**	**0.306**	**0.2346**	**C/T**	**Phe153Leu**	**B/T**	**0.020456**	**rs1132185**	***C1orf158***
1	156267524	1q22	0.000	0.139	0.01	T/C	Ile355Val	B/T	0.0227273	rs145100575	*SMG5*
11	5986042	11p15.4	0.083	0.250	0.1899	G/A	Trp297Arg	B/D	0.0375179	rs4237768	*OR52L1*
17	37624364	17q12	0.278	0.111	0.2469	C/T	Met403Val	B/T	0.0429011	rs7216445	*DDX52*
19	44477666	19q13.31	0.333	0.472	0.4727	G/C	Cys272Ser	B/T	0.0438416	rs1897820	*ZNF180*
19	44497294	19q13.31	0.361	0.500	0.4932	A/G	Ala41Val	B/T	0.0227273	rs2571108	*ZNF180*
**19**	**54664811**	**19q13.42**	**0.139**	**0.333**	**0.3021**	**G/A**	**Met113Val**	**B/D**	**0.021855**	**rs731170**	***LILRB4***
**19**	**54818581**	**19q13.42**	**0.278**	**0.111**	**0**	**G/A**	**Glu144Ala**	**B/D**	**0.042901**	**rs643861**	***KIR3DL1, KIR3DS1***
**HLA–**											
8	144379425	8q24.3	0.333	0.472	0.4882	C/A	Arg17Ser	B/T	0.0438416	rs6599528	*ADCK5*
22	22514169	22q11.22	0.333	0.167	0.2745	T/G	Arg488Ser	B/D	0.0471713	rs361666	*ZNF280A*
22	22514173	22q11.22	0.333	0.167	0.2745	C/G	Phe486Leu	B/T	0.0471713	rs361762	*ZNF280A*
22	22514885	22q11.22	0.333	0.167	0.2745	G/C	Gly249Ala	B/T	0.0471713	rs362124	*ZNF280A*
22	22514894	22q11.22	0.333	0.167	0.2745	C/T	Asn246Ser	B/T	0.0471713	rs362132	*ZNF280A*
22	22515221	22q11.22	0.333	0.167	0.275	G/T	Tyr137Ser	B/T	0.0471713	rs361580	*ZNF280A*
22	22515224	22q11.22	0.333	0.167	0.2753	C/T	Asn136Ser	B/T	0.0471713	rs362011	*ZNF280A*
22	22515418	22q11.22	0.333	0.167	0.2742	A/C	Lys71Asn	B/T	0.0471713	rs361959	*ZNF280A*

*Bolded are the chromosomal locations were muliple variants were associated*.

*Resolved patients, n = 36; persitent patients, n = 36; HLA+ resolved, n = 18; HLA+ persistent, n = 18; HLA− resolved, n = 18, HLA− persistent, n = 18*.

*Predicted function according to PolyPhen and Sift, respectively: B, benign; P, probably damaging; T, tolerated; D, deleterious. MAF, minor allele frequency; gnomAD, The Genome Aggregation Database; AA, amino acid. P values are uncorrected, none of the values remained significant after correcting for multiple tests*.

### Gene-Based Analysis

In gene-based tests, all variants found in the same gene are grouped to assess the association with the phenotype. Also, in gene-based analysis the locations 1p36.21 and 19q13.42 showed associations while not remained significant after correcting with FDR or Bonferroni correction (Table 2). In location 19q13.42, variants in gene *LAIR1* were also significantly associated with the prognosis, in addition to previously seen *LILRB4* and *KIR3DL1/KIRSDS1*. Manhattan plot from the gene-based analysis between HLA+ persistent and resolved patients is seen in Supplementary Figure 2. No association in locations 1p36.21 and 19q13.42 was seen in HLA- patients. Another chromosomal location was also seen: in 2p22.3, two genes were shown to associate with disease prognosis; *RASGRP3* with persistent disease and *LTBP1* in resolved disease. However, these genes did not show significant association in any of the other subgroup analysis.

**Table 2:** Associated genes in gene-based analysis (hypergeometric distribution and SKAT-O methods) in different Finnish sarcoidosis subgroups based on disease prognosis (persistent, resolved) and HLA markers (HLA+ = HLA-DRB1^*^03:01 and/or HLA-DRB1^*^04:01-DPB1^*^04:01; HLA– = without either of the markers).

**Chromosome**	**Region**		***P* HGD**	***P* SKAT-O**	**Gene**	**Associated trait**
**PERSISTENT VS. RESOLVED**						
1	109252166–109273493	1p13.3	0.012466754	0.021725009	*CELSR2*	Persistent
**1**	**12716215–12725527**	**1p36.21**	**0.04489705**		***AADACL3***	Resolved
**1**	**12755700–12760937**	**1p36.21**	**0.00429823**		***C1orf158***	Resolved
1	156267524–156267524	1q22	0.026944274	0.021343706	*SMG5*	Resolved
1	23519074–23521037	1p36.12	0.012466754	0.024629574	*E2F2*	Resolved
2	178532055–178781235	2q31.2	0.028053232		*TTN*	Resolved
**2**	**32947681–33342904**	**2p22.3**	**0.02694427**		***LTBP1***	Resolved
**2**	**33524504–33527173**	**2p22.3**	**0.02694427**	**0.03932876**	***RASGRP3***	Persistent
8	144467535–144469561	8q24.3	0.026944274	0.039865155	*KIFC2*	Resolved
9	128990198–129005527	9q34.11	0.026944274	0.039865155	*NUP188*	Persistent
11	20160309–20160309	11p15.1		0.040988976	*DBX1*	Persistent
12	52692557–52692557	12q13.13		0.040988976	*KRT77*	Persistent
19	17102004–17212090	19p13.11	0.031601364	0.034412198	*MYO9B*	Persistent
19	42079904–42080806	19q13.2	0.026944274	0.039865155	*ZNF574*	Persistent
**19**	**54361091–54361091**	**19q13.42**		**0.04825453**	***LAIR1***	Resolved
**19**	**54664811–54664811**	**19q13.42**	**0.01644974**		***LILRB4***	Resolved
**19**	**54818479–54819917**	**19q13.42**	**0.04676604**		***KIR3DL1, KIR3DS1***	Persistent
22	27798761–27800302	22q12.1	0.012466754	0.0183	*MN1*	Persistent
22	32491131–32498513	22q12.3	0.026944274		*FBXO7*	Persistent
**HLA+**						
**1**	**12716215–12725527**	**1p36.21**	**0.01770751**		***AADACL3***	Resolved
**1**	**12755700–12760937**	**1p36.21**	**0.00879604**		***C1orf158***	Resolved
1	156267524–156267524	1q22	0.022727273		*SMG5*	Resolved
3	12004712–12004864	3p25.2	0.04290113		*SYN2*	Resolved
11	5986042–5986042	11p15.4	0.037517877		*OR52L1*	Resolved
17	37624364–37628587	17q12	0.04290113		*DDX52*	Persistent
19	44477645–44497294	19q13.31	0.022727273		*ZNF180*	Resolved
**19**	**54664811–54664811**	**19q13.42**	**0.02185508**		***LILRB4***	Resolved
**19**	**54818479–54819917**	**19q13.42**	**0.04290113**		***KIR3DL1, KIR3DS1***	Persistent
**HLA–**						
2	178532055–178781235	2q31.2	0.020455901		*TTN*	Resolved
8	10609233–10623110	8p23.1	0.022727273	0.03576884	*RP1L1*	Resolved
8	144379425–144392522	8q24.3	0.043841642		*ADCK5*	Resolved
19	17102004–17212090	19p13.11	0.022727273	0.03576884	*MYO9B*	Persistent
22	22514169–22515418	22q11.22	0.047171298		*ZNF280A*	Persistent

*Bolded are the chromosomal locations were multiple genes are associated*.

Resolved patients, n = 36; persitent patients, n = 36; HLA+ resolved, n = 18; HLA+ persistent, n = 18; HLA– resolved, n = 18, HLA– persistent, n = 18.

HGD, hypergeometric distribution method.

*P values are uncorrected, none of th values remained significant after correcting for multiple tests*.

### Replication Analysis

For the replication analysis, the rest of the Finnish sample set of 188 samples and 150 Finnish control material were included. After quality control, total of 150 controls and 181 cases (72 from the WES study and 109 from the replication study) were included in the analysis. In the replication analysis between persistent and resolved sarcoidosis patients, the SNPs in the chromosomal location 1p36.21 were statistically significant as in the WES study (Table 3). The association in these SNPs was also found in the analysis between HLA + persistent and resolved patients, and in contrast to the WES study, these SNPs were also significantly associated in the HLA-disease subgroup. When combining the data from the WES and replication studies, the associations became even stronger in all subgroup analysis. In chromosomal location 19q13.42, *KIR3DL1/KIR3DS1* SNPs did not replicate and *LILRB4* SNPs associated significantly in the HLA + resolved patients in the combined data set (Table 3). None of the associations remained statistically significant after correction with SNP count (data not shown). However, the MAFs in the control population corresponded to the ones in the gnomAD Finnish control population except for the *KIR3DL1/KIR3DS1* SNP rs643861, which lacked allele frequency data in the gnomAD database (Table 3). However, in the NCBI dbSNP database (https://www.ncbi.nlm.nih.gov/snp/) MAF from TopMed database is A = 0.185, which resembles MAF found in our study. TOPMed uses hg38 as a reference genome, in gnomAD the hg19 is used as a reference genome which might explain the differences.

**Table 3:** Replication of SNPs found in the preliminary Whole-exome sequencing analysis in different Finnish sarcoidosis subgroups based on disease prognosis (persistent, resolved) and HLA markers (HLA+ = HLA–DRB1^*^03:01 and/or HLA–DRB1^*^04:01-DPB1^*^04:01; HLA– = without either of the markers) and Finnish control population.

**Chromosome**	**Position**	**Chromosomal location**	MAF^*^ Persistent	MAF^*^ Resolved	**MAF gnomAD Finnish**	**MAF Control**	MAF replication^**^ Persistent	MAF replication^**^ Resolved	MAF combined^***^ Persistent	MAF combined^***^ Resolved	**SNP**	***P***	***P* Replication**	***P* Combined**	**rsID**	**Gene**
**PERSISTENT VS. RESOLVED**																
1	12719616	1p36.21	0.125	0.375	0.2196	0.2166	0.1667	0.2885	0.1505	0.3239	C/T	0.000915	0.03142	0.0001009	rs3010877	*AADACL3*
1	12725527	1p36.21	0.181	0.389	0.3072	0.2866	0.2281	0.375	0.2097	0.3807	T/G	0.0165913	0.01785	0.0003522	rs7513079	*AADACL3*
1	12760937	1p36.21	0.139	0.361	0.2346	0.2261	0.1667	0.3173	0.1559	0.3352	C/T	0.0083299	0.009143	7.031E-05	rs1132185	*C1orf158*
19	54664811	19q13.42	0.208	0.375	0.3021	0.2834	0.3421	0.3558	0.2903	0.3636	G/A	0.0164497	0.8325	0.1369	rs731170	*LILRB4*
19	54818581	19q13.42	0.264	0.153	0	0.2677	0.2544	0.2692	0.2796	0.233	G/A	0.046766	0.8033	0.3103	rs643861	*KIR3DL1, KIR3DS1*
**HLA+**																
1	12719616	1p36.21	0.083	0.361	0.2196	0.2166	0.1071	0.22	0.09375	0.314	C/T	0.0010228	0.2128	5.27E-06	rs3010877	*AADACL3*
1	12725527	1p36.21	0.111	0.333	0.3072	0.2866	0.1071	0.32	0.1094	0.3721	T/G	0.008796	0.03565	2.88E-07	rs7513079	*AADACL3*
1	12760937	1p36.21	0.111	0.306	0.2346	0.2261	0.07143	0.26	0.09375	0.3256	C/T	0.0204559	0.04265	2.10E-06	rs1132185	*C1orf158*
19	54664811	19q13.42	0.139	0.333	0.3021	0.2834	0.3929	0.42	0.25	0.407	G/A	0.0218551	0.8152	0.004529	rs731170	*LILRB4*
19	54818581	19q13.42	0.278	0.111	0	0.2677	0.25	0.3	0.2812	0.2674	G/A	0.0429011	0.6378	0.8511	rs643861	*KIR3DL1, KIR3DS1*
**HLA–**																
1	12719616	1p36.21	0.1667	0.3056	0.2196	0.2166	0.186	0.3519	0.1803	0.3333	C/T	0.1653	0.02743	0.01049	rs3010877	*AADACL3*
1	12725527	1p36.21	0.25	0.3333	0.3072	0.2866	0.2674	0.4259	0.2623	0.3889	T/G	0.4367	0.05197	0.05005	rs7513079	*AADACL3*
1	12760937	1p36.21	0.1667	0.3056	0.2346	0.2261	0.1977	0.3704	0.1885	0.3444	C/T	0.1653	0.02409	0.01001	rs1132185	*C1orf158*

* *Resolved patients, n = 36; persistent patients, n = 36; HLA+ resolved, n = 18; HLA+ persistent, n = 18; HLA– resolved, n = 18, HLA– persistent, n = 18*.

** Resolved patients, n = 52; persistent patients, n = 57; HLA+ resolved, n = 25; HLA+ persistent, n = 14; HLA– resolved, n = 27, HLA– persistent, n = 43.

*** Resolved patients, n = 88; persistent patients, n = 93; HLA+ resolved, n = 43; HLA+ persistent, n = 32; HLA– resolved, n = 45, HLA– persistent, n = 61. Predicted function according to PolyPhen and Sift, respectively: B, benign; P, probably damaging; T, tolerated; D, deleterious. *MAF, minor allele frequency; AA, amino acid*.

The WES study associations were found in single-variation and gene levels in the chromosomal locations 1p36.21 and 19q13.42. In the replication sample, the associations in the location 1p36.21 were found only in the SNPs associating in the single-variation analysis (Supplementary Table 3). In chromosomal location 19q13.42, none of the other SNPs in the *KIR3DL1/KIR3DS1* associated with the disease (data not shown). *LAIR1* and *KIR3DL3* SNPs did not meet the Sequenom study design criteria and could not be replicated (Supplementary Table 3). In chromosomal location 2p22.3, two genes were shown to associate with the disease prognosis in the WES study; *RASGRP3* with persistent disease and *LTBP1* in resolved disease. In the replication study, only one SNP in the *RASGRP3* and *LTBP1* passed the quality control (Supplementary Table 3) and none of these SNPs associated with the trait (data not shown).

## Discussion

The heterogeneity of sarcoidosis in clinical course and development of organ involvement suggests that manifestation of the disease phenotype is a result of multiple genetic variations. There is a special interest in finding genetic differences between sarcoidosis patients with different prognosis, because at the moment prediction of the disease course at the time of diagnosis is difficult. There are also significant ethnic differences in disease development and prognosis in different populations (19). These differences represent a compelling need for ethnic-selective biomarkers to assess disease progression in diverse populations.

Here we present results from WES study in Finnish sarcoidosis patients. The strategy was to sequence groups of sarcoidosis patients with known differences in prognosis to identify variants that could alter functional properties of proteins. In Finnish sarcoidosis patients, certain HLA markers (HLA-DRB1*03:01, HLA-DRB1*04:01-DQB1*04:01) have been shown to be more common in patients with sarcoidosis disease resolving within 2 years, i.e., good prognosis disease. The aim of this study was to further pinpoint genetic variety in different sarcoidosis prognosis groups in relation to these HLA markers.

The most strongly associated and replicated chromosomal location was found in 1p36.21, containing two genes (*AADACL3* and *C1orf158*). This region has also been found to associate with familial sarcoidosis in a recent WES study in German patients (20). However, in French WES study of familial sarcoidosis, no association with this region was found (21). In our study, variations in these genes were associated with disease resolution independent of the HLA markers DRB1*03:01 and DRB1*04:01. The found variations in *AADACL3* are predicted to be benign, but the variation in *C1orf158* is thought to alter the function of the protein. However, the functionality of these variants in sarcoidosis predisposition is unclear at the moment. The variants rs3010877 in *AADACL3* and rs1132185 in *C1orf158* are known to act as eQTLs in not sun exposed skin, but no clear in sarcoidosis pathogenesis is evident. Due to LD, it is also possible that association derives from elsewhere in the chromosome. However, no functionally plausible genes reside in near proximity of these genes, while the further region in 1p36 has been associated with resolution of chest findings in sarcoidosis patients (22). Therefore, further studies are needed to evaluate the possible causality of these genes and sarcoidosis susceptibility.

In the WES study, another interesting chromosomal region was seen in 19q13.42 (*LILRB4, KIR3DL1/KIR3DS1*, and *LAIR1*). In the replication part of the study, only association with *LILRB4* remained statistically significant when WES and replication data were combined, the other associations were weakened or could not been replicated. It should be noted, that the size of the replication material is still relatively small, and possible true causality cannot be ruled out.

The 19q13.42 region, called the Leukocyte Receptor Complex (LRC), comprises a large set of genes encoding immunoglobulin superfamily receptors (23). The leukocyte immunoglobulin-like receptors (LILR) can interact with the HLA class I (24) and are expressed by a range of immunologically active cells, including natural killer (NK) and antigen-presenting cells (APCs) (25). LILRs have both activating and inhibitory effects (26). Previously no associations have been found between LILRB4 and sarcoidosis, but variations in this gene have shown to increase risk of another immune-mediated disease, systemic lupus erythematosus (SLE) (27). In our study, variations in this gene associated with good prognosis patients having the good prognosis HLA Class II markers. *LILRB4* belongs to inhibitory receptors expressed on dendritic cells and has been shown to interact with the HLA-G. (28). LILRB4-HLA-G interaction has been demonstrated to limit the activation of dendritic cells (29), which are important APCs in sarcoidosis inflammation. In sarcoidosis, the association with HLA-II is thought to be more important, but it should be noted that limited information exists in relation to HLA-G in sarcoidosis. In one study, the HLA-G variation was seen in sarcoidosis patients (30). Also, there is a strong LD in the MHC region, and it is possible that patients with the HLA class II markers have haplotypes containing the HLA class I as well. The combination of these MHC variations, with the variants in the *LILRB4*, could alter the immune reaction in sarcoidosis and lead to self-resolving disease.

In addition to *LILRB4*, in the WES study, association was found with variants in *LAIR1* and *KIR3DL1/KIR3DS1*. Leukocyte-associated immunoglobulin-like receptor-1 (*LAIR1*) has been shown to inhibit T and NK cell activation (31), but there are no known HLA ligands for this receptor. In our study, association with variants in the *LAIR1* was seen when persistent and resolved patients were compared. No association was evident with the HLA markers, which is in concordance with the notion that there are no known HLA ligands for this protein. No association have been found between this gene and sarcoidosis, but also this immune-modulator gene has been shown to have effect in SLE (32). However, this association was seen only in gene-based analysis and with single method. Due to technical issues, the variation could not be replicated, so the effect should be considered cautiously. The other association seen only in the WES study encode KIRs, receptors expressed mainly on the surface of natural killer (NK) cells. Most diverse of the *KIR* genes is locus *KIR3DL1/KIR3DS1* which associated with persistent sarcoidosis in our study. The gene encodes both inhibitory (3DL1) and activating (3DS1) receptors (33), so either or both of these functions could be affected, shifting the course of the disease toward chronic. In a recent WES study of three families sharing pediatric sarcoidosis, the *KIR3DL1/KIR3DS1* in gene level was found to be shared in these families (34). To our knowledge, no case-control study has found association in *KIR* genes and sarcoidosis.

There are some limitations in this study. Sarcoidosis being a relatively rare disease, the collection of large data sets is demanding in a small country as Finland. Therefore, due to relatively small sample size, the power might have not been sufficient to detect all associations, however, the gene-based analysis in the WES study and the replication sample increases the power. Because of the patient selection based on the HLA markers, the analysis between all persistent and resolved patients has some statistical limitations. However, in the replication study, the whole Finnish sarcoidosis sample is involved. The other confounding factors: gender, age and extrapulmonary manifestations, were conditioned in all analyses.

In summary, we used WES to further characterize genetic differences between persistent and resolved sarcoidosis patients and to test the hypothesis whether different variations are associated in relation to the class II markers (HLA-DRB1*03:01/HLA-DRB1*04:01-DPB1*04:01). An association with resolved disease raised in the chromosomal region 1p36.21, a region which has recently been associated with sarcoidosis in another WES study. Another interesting chromosomal region peaked, 19q13.42, but in the replication study the association weakened. However, due to relatively small sample size, the true causality of these variants should be evaluated in a larger data set.

## Data Availability Statement

The raw data supporting the conclusions of this article will be made available by the authors, without undue reservation, to any qualified researcher.

## Ethics Statement

The studies involving human participants were reviewed and approved by the Ethics Committee of the Department of Internal Medicine, in the Hospital District of Helsinki and Uusimaa, Helsinki, Finland (Approval Dnro 362/E5/05). The patients/participants provided their written informed consent to participate in this study.

## Author Contributions

EL, MK, JS, OS, and M-LL: study design and data analysis and manuscript writing. The manuscript has been read and approved by all named authors.

### Conflict of Interest

The authors declare that the research was conducted in the absence of any commercial or financial relationships that could be construed as a potential conflict of interest.
