# Supplementary material for: Exome Sequencing Identifies Susceptibility Loci for Sarcoidosis Prognosis
Source: Front Immunol. 2019 Dec 24;10:2964. doi: 10.3389/fimmu.2019.02964 (PMC6937869; doi:10.3389/fimmu.2019.02964)
Supplement: Supplementary file 1 [file Data_Sheet_1.docx]

Supplementary Material

# Supplementary Figures and Tables

## Supplementary Tables

**Supplementary Table 2.** Version information and references of tools used in variant calling in whole-exome sequencing analysis.

1. Bolger, A. M., Lohse, M. & Usadel, B. Trimmomatic: a flexible trimmer for Illumina sequence data. Bioinformatics 30, 2114–2120 (2014).

2. FastQC: a quality control tool for high throughput sequence data. Available online at: http://www.bioinformatics.babraham.ac.uk/projects/fastqc

3. Li, H. Aligning sequence reads, clone sequences and assembly contigs with BWA-MEM. arXiv (2013).

4. McKenna, A. et al. The Genome Analysis Toolkit: a MapReduce framework for analyzing next-generation DNA sequencing data. Genome Res. 20, 1297–1303 (2010).

5. Picard-Tools: <http://picard.sourceforge.net>

6. Wang K, Li M, Hakonarson H. ANNOVAR: functional annotation of genetic variants from high-throughput sequencing data. Nucleic Acids Res 2010 Sep;38(16):e164.

7. Kang HM. 2012. EPACTS: efficient and parallelizable association container toolbox. Department of Biostatistics and Center for Statistical Genetics, University of Michigan. Available at: <http://www.sph.umich.edu/csg/kang/epacts/>.

**Supplementary Table 3.** List of single nucleotide polymorphisms (SNPs) genotyped in the replication Finnish samples. SNPs marked in light grey did not meet the Sequenom study design criteria and SNPs marked in dark grey did not meet the quality control criteria (minimum call rate per sample of 90 %, minor allele frequency (MAF) > 0.01, Hardy Weinberg equilibrium (HWE) > 0.001).

##
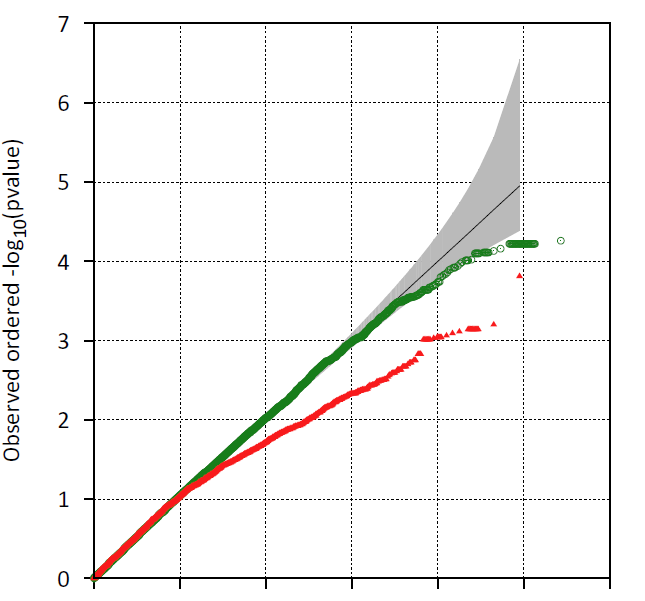
Supplementary Figures

**Supplementary Figure 1.** The quantile-quantile (Q-Q) plot for the single-variant tests between persistent (green) and resolved (red) patients. This Q-Q plot shows observed (y axis) versus expected (x axis) ordered −log10 p-values with the shaded region showing standard errors.


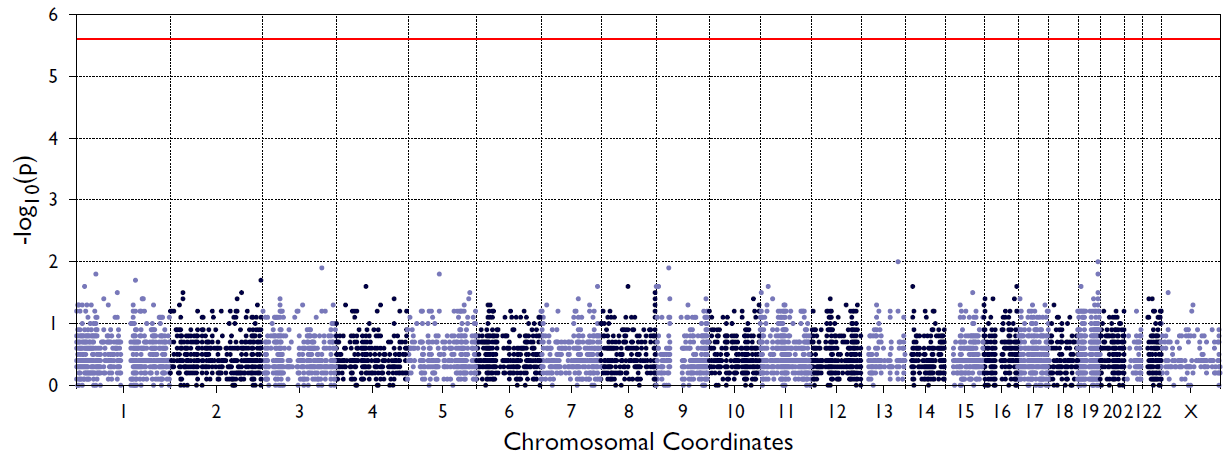


**Supplementary Figure 2.** Manhattan plot from the single-variant association tests between persistent and resolved patients with Class II HLA markers (*HLA-DRB1*03:01* and/or *HLA-DRB1*04:01-DPB1*04:01* haplotype). The X-axis shows chromosome position the Y-axis shows the negative log of p-values so that higher values represent stronger significance levels. The horizontal line shows the threshold value for significance.
